# Training initiatives within the AFHSC-Global Emerging Infections Surveillance and Response System: support for IHR (2005)

**DOI:** 10.1186/1471-2458-11-S2-S5

**Published:** 2011-03-04

**Authors:** Jean L Otto, Priya Baliga, Jose L Sanchez, Matthew C Johns, Gregory C Gray, John Grieco, Andres G Lescano, Jerry L Mothershead, Eric J Wagar, David L Blazes, the AFHSC-GEIS Training Writing Group

**Affiliations:** 1 Armed Forces Health Surveillance Center, 11800 Tech Rd, Silver Spring, MD 20904, USA; 2 Department of Environmental and Global Health, College of Public Health and Health Professions University of Florida, Post Office Box 100188, Gainesville, FL 32610, USA; 3 Uniformed Services University of the Health Sciences, 4301 Jones Bridge Road, Bethesda, MD 20814, USA; 4 Naval Medical Research Center Detachment, Centro Medico Naval “CMST,” Av. Venezuela CDRA 36, Callao 2, Lima, Peru; 5 Center for Disaster and Humanitarian Assistance Medicine, Uniformed Services University of the Health Sciences, F. Edward Hébert School of Medicine, 4301 Jones Bridge Road, Bethesda, MD 20814, USA; 6 U.S. Embassy, Attention: MRU, United Nations Avenue, Post Office Box 606, Village Market 00621 Nairobi, Kenya; 7 Makerere University Walter Reed Project, Influenza Research Programme, Post Office Box 7062, Kampala, Uganda; 8 University of Iowa Center for Emerging Infectious Diseases, 2501 Crosspark Road, MTF B145, Coralville, Iowa 52241, USA; 9 Naval Health Research Center, 140 Sylvester Road, San Diego, CA 92106, USA; 10 Medical Brigade/USAMEDDAC-Korea, Unit 15281, APO AP 96205-5281, USA; 11 Public Health Region-Europe, CMR 402, APO AE 09180, USA; 12 Navy Environmental Preventive Medicine Unit 2, 1887 Powhatan Street, Norfolk, VA 23511-3394, USA; 13 Pacific Air Forces, 990 Scott Circle, Hickam Air Force Base, HI 96853, USA; 14 U.S. Air Force School of Aerospace Medicine, Epidemiology Consult Service, 2513 Kennedy Circle, Building 180, Brooks City Base, TX 78235, USA; 15 Naval Medical Research Unit Number 3, Extension of Ramses Street, Adjacent to Abbassia Fever Hospital, Postal Code 11517, Cairo, Egypt; 16 University of Buea, Department of Biochemistry and Microbiology, Faculty of Science, Post Office Box 63, Buea, South Western Province, Cameroon; 17 Armed Forces Research Institute of Medical Sciences, 315/6 Rajavithi Road, Bangkok, Thailand 10400; 18 Public Health Region-South, Building 2472 Schofield Road, Fort Sam Houston, TX 78234, USA; 19 Naval Medical Research Unit Number 2, Kompleks Pergudangan DEPKES R.I., JI. Percetakan Negara II No. 23, Jakarta, 10560, Indonesia

## Abstract

Training is a key component of building capacity for public health surveillance and response, but has often been difficult to quantify. During fiscal 2009, the Armed Forces Health Surveillance Center, Division of Global Emerging Infections Surveillance and Response System (AFHSC-GEIS) supported 18 partner organizations in conducting 123 training initiatives in 40 countries for 3,130 U.S. military, civilian and host-country personnel. The training assisted with supporting compliance with International Health Regulations, IHR (2005). Training activities in pandemic preparedness, outbreak investigation and response, emerging infectious disease (EID) surveillance and pathogen diagnostic techniques were expanded significantly. By engaging local health and other government officials and civilian institutions, the U.S. military’s role as a key stakeholder in global public health has been strengthened and has contributed to EID-related surveillance, research and capacity-building initiatives specified elsewhere in this issue. Public health and emerging infections surveillance training accomplished by AFHSC-GEIS and its Department of Defense (DoD) partners during fiscal 2009 will be tabulated and described.

## Background

In order to achieve optimal and coordinated implementation of IHR (2005), each member state must be capable of detecting, confirming, reporting and containing an emerging threat to public health [1] by 2012. To reach these 2012 milestones, member states will need to significantly enhance their laboratory infrastructure, and more importantly, appropriately train personnel who can perform the core-capacity functions defined in Article 5 of IHR (2005). These core capacities include leading outbreaks investigations, correctly identifying a pathogen in the laboratory, rapidly communicating findings to stakeholders at all levels, and most importantly, controlling the outbreak through tested and exercised mitigation efforts.

Front-line public health professionals require the latest knowledge and skills to address the evolving nature of potential global threats to public health. The recent pandemic of novel influenza A/H1N1 clearly illustrates the unpredictable nature of pathogens that require dynamic and evolving public health strategies for surveillance, disease management and mitigation.

Training public health professionals, both host-country and U.S. DoD personnel, to understand, monitor, respond to, control and prevent emerging infections is a foundational goal of AFHSC-GEIS. The center has the mission of conducting surveillance for emerging infectious diseases that could affect the U.S. military [2]. AFHSC-GEIS promotes national and international preparedness for emerging infections through orchestrating a global array of surveillance projects, capacity-building efforts, outbreak investigations and training exercises [2].

Since its inception, AFHSC-GEIS partners and collaborators have made available their overseas laboratory and field study facilities to serve as regional focal points for the training of staff, technicians and epidemiologists within partner host countries [3-5] through a growing collaborative network of U.S. government agency partners. A wide range of training has been coordinated at these sites, including programs such as the Centers for Disease Control and Prevention’s (CDC’s) Field Epidemiology Training and Laboratory Training Programs [6] and the U.S. Agency for International Development efforts through Africa and the Pacific [7,8]. These training opportunities serve as a forum for support, coordination and collaboration with host-country partners as prescribed in Article 44 of the IHR (2005). These efforts are conducted in close collaboration with host-country counterparts utilizing standardized teaching tools and guidelines developed by WHO [9] and other international global health entities to enhance core public health capacities within each partner host country.

In addition, through the Uniformed Services University of the Health Sciences (USUHS) Center for Disaster and Humanitarian Assistance Medicine (CDHAM), educational efforts in support of five combatant commands have been bolstered with AFHSC-GEIS funding. Such training provides much-needed professional expertise and the latest technical information to U.S. military and civilian health care providers, as well as to host-country Ministries of Health, Agriculture and Defense, and other civilian agency collaborators. These initiatives result in significantly improving the professional engagement of host-country officials, as well as further enhancing the U.S. government’s role as a key stakeholder in the global health community. Training initiatives encompass such topics as planning and preparedness, outbreak investigation, surveillance and response for a wide spectrum of disease- or syndrome-causing agents. This report summarizes training initiatives and highlights select training projects conducted by AFHSC-GEIS-funded partners from September 2008 to October 2009.

## Methods

Authors of this report reviewed all fiscal year 2009 (September 2008 to October 2009) individual project reports, as well as program reports from AFHSC-GEIS coordinators submitted to AHFSC-GEIS, and identified specific training programs conducted by partners. In this review, the authors included training activities such as workshops, academic courses, conferences, tabletop exercises and distance learning. Stand-alone lectures, descriptive program presentations and poster presentations are not included, although these remain an integral part of disseminating program findings and information.

## Results

During fiscal 2009, 18 partner organizations conducted 123 training initiatives in 40 countries, reaching approximately 3,130 U.S. military and civilian personnel, as well as host-nation personnel (Additional file 1 and Table 1). These educational efforts covered a wide range of topics. The most common categories were EID laboratory techniques (41 percent), pandemic influenza (24 percent) and disease surveillance techniques (19 percent). Training modalities included workshops (defined here as hands-on, interactive training), academic courses, conferences, tabletop exercises, and on-line and telephonic distance learning. Duration of training ranged from 0.5 hours to six months, with the majority of education efforts lasting two to five days.

**Table 1:** AFSHC-GEIS-Funded Training Initiatives by Combatant Command Location of Training, October 2008-September 2009

Combatant Command	No. total training initiatives	No. countries	No. trainees*
U.S. Africa Command	15	10	294
U.S. Central Command	17	6	454
U.S. European Command	19	8	1057
U.S. Northern Command	28	1	595
U.S. Pacific Command	35	10	623
U.S. Southern Command	9	5	107
TOTAL	123	40	3130

*Where exact figures are not known, an estimate of the number of trainees is provided.

**Figure 1:**
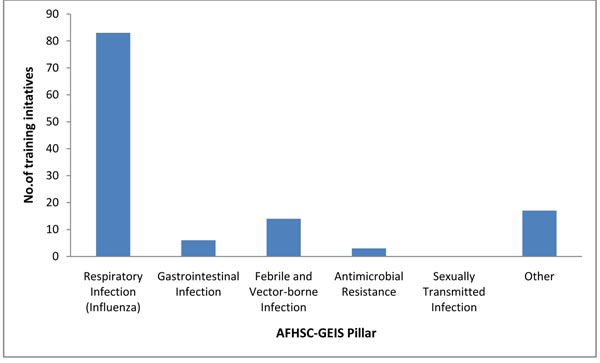
AFHSC-GEIS-Funded Training Initiatives by AFHSC-GEIS Priority Pillar, October 2008-September 2009

**Figure 2:**
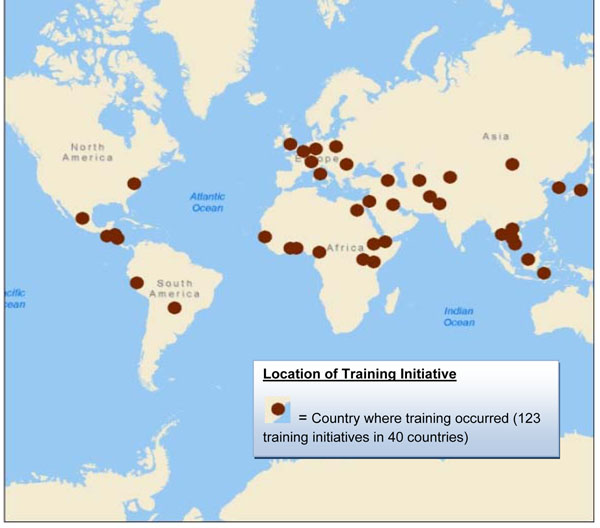
Geographic Distribution of AFHSC-GEIS-Funded Training Initiatives, October 2008-September 2009

In terms of AFHSC-GEIS pillars, respiratory infections (namely influenza) represented the majority of training initiatives (68 percent), followed by febrile and vector-borne infections (11 percent), gastrointestinal infections (5 percent), and antimicrobial resistance (2 percent). The remaining 14 percent included various topics (geographic information systems, EpiInfo, field epidemiology, principles and practice of clinical research, outbreak investigation, emerging infectious diseases, lab safety precautions, ecological niche modeling and tropical medicine student rotations) (Figure 1). Training initiatives conducted in fiscal 2009 did not focus on sexually transmitted infections. Training occurred in each of the six Combatant Commands (COCOM). The most occurred in the U.S. Pacific Command (USPACOM) and the least in U.S. Southern Command (USSOUTHCOM) (Table 1). Figure 2 shows the geographic distribution of these initiatives across the globe.

The following sections describe selected examples of training initiatives that highlight the breadth of AFHSC-GEIS efforts in this arena.

## CDHAM combatant command training

All U.S. military initiatives are coordinated by six COCOMs specific to geographic regions of the world: USPACOM, U.S. European Command (USEUCOM), U.S. Northern Command (USNORTHCOM), U.S. Central Command (USCENTCOM), U.S. Southern Command (USSOUTHCOM) and U.S. Africa Command (USAFRICOM). These geographic COCOMs provide U.S. military representation to international and U.S. national agencies within their area of responsibility (AOR).

In fiscal 2009, AFHSC-GEIS funded training programs for COCOMs through CDHAM. Affiliated with USU, CDHAM assisted COCOMs in developing, planning and executing education and training programs for pandemic and avian influenza surveillance and response. As depicted in Additional file 1, in fiscal 2009, CDHAM conducted a total of 12 conferences and tabletop exercises, training 673 individuals in support of four COCOMs: USPACOM, USEUCOM, USCENTCOM and USNORTHCOM. CDHAM conducted two training initiatives in the USPACOM’s area of responsibility: a workshop on public health management of pandemic influenza (PHMPI) in Malaysia and the USPACOM Medical Surge Conference, held in Indonesia, which drew 64 participants from India and Indonesia. In EUCOM, CDHAM supported four separate PHMPI workshops in Armenia, Poland, Romania and the United Kingdom involving approximately 190 participants, as well as a European pandemic influenza (PI) Synchronization Conference in Switzerland for 55 participants.

In support of USCENTCOM, CDHAM planned and executed two PHMPI workshops in Kyrgyzstan, which involved 60 participants from Tajikistan, Turkmenistan, the United States and Uzbekistan; and one in Pakistan for 40 participants. These workshops offered a venue for each of the country’s Ministries of Defense to present their respective PI plan, focusing on strategies of containment, identifying available resources and existing gaps, and identifying and addressing these gaps with the assistance of the U.S. DoD. The program for this conference comprised three days of lectures, country briefs and breakout sessions.

## University of Iowa certificate in emerging infectious disease epidemiology

Developing countries have a strong desire to acquire advanced training in public health from recognized universities in the U.S. and Europe. Many AFHSC-GEIS partners are asked to support this type of training. The University of Iowa developed a certificate program to provide training at an internationally known U.S. university, through graduate level coursework that trainees could use as an entry to additional graduate studies and potentially the completion of a master of public health (M.P.H.) degree. The certificate program aims to build sustainable epidemiology research capacity in infectious disease by promoting collaborations between international U.S. laboratories and other nations. The program also trained promising young public health officials who will hopefully support public health capacity in their home countries and contribute to the control of local epidemics that may have a global effect.

With AFHSC-GEIS funding, the University of Iowa’s College of Public Health created a 12-semester-hour Certificate in Emerging Infectious Disease Epidemiology, nine hours of which may be applied toward an M.P.H. degree. For the summer 2009 class, 33 foreign nationals (from Bangladesh, Egypt, Indonesia, Kenya, Mongolia, Nepal, Nigeria, Pakistan, Panama, Peru, the Philippines, Poland, Rwanda and Thailand) were nominated and enrolled in the certificate program by the DoD overseas research laboratory commanders, AFHSC-GEIS staff, CDC, and U.S. Department of State. Intensive training was conducted in Iowa City for two weeks (six semester hours) that included lectures, tutorials, field experiences, lab exercises, public health demonstrations and written examinations. The remaining credits were earned via web-based curricula. [10]

## U.S. Army Medical Research Unit-Kenya (USAMRU-K) Malaria Diagnostics Center of Excellence

The continued operation of the Malaria Diagnostics Center of Excellence (MDCoE) in Kisumu, Kenya, provided important contributions in professional malaria diagnostic training. The MDCoE was established in 2004 with AFHSC-GEIS funding. Objectives of the center include training microscopists working in the clinical setting in developing countries and transferring technology to host countries [11].

In fiscal 2009, the MDCoE conducted seven basic malaria microscopy courses, four of which were AFHSC-GEIS-funded, which reached 55 laboratory technicians from Kenya, Nigeria, Republic of South Africa, Tanzania, Uganda and the United States. Each course consisted of 10 days of laboratory practical sessions, lectures, group discussions, demonstrations, take home assignments and pre- and post-course examinations.

By training microscopists who support malaria clinical trials and disease surveillance projects, the MDCoE helped to improve the quality of data generated by these projects. In addition, training of Kenyan clinical microscopists, as well as microscopists from other African countries, will hopefully improve the quality of health care delivery and potentially contribute to a reduction in the malaria disease burden in the local population.

The MDCoE also conducted a mosquito taxonomy and control course, which was taught to 15 Kenyans from the Division of Vector-Borne Diseases in February 2009. This course focused on insect vector taxonomy, identification and control. By teaching this course, USAMRU-K was better able to understand Kenyan vector-control efforts and learn of locations with high risks for vector-borne diseases. They were also able to better identify potential sites for future surveillance activities. The addition of this course expanded outreach potential and should lead to improvement of the quality of their vector surveillance and control programs in Africa.

## USUHS–geographic information systems

In the fields of public health and epidemiology, geographic information systems (GIS) have become increasingly popular and beneficial as a tool for tracking epidemics and showing geographic trends of various diseases. In fiscal 2009, USUHS offered important training in the use of GIS for military and civilian students, as well as overseas laboratory personnel.

The University developed and conducted five basic GIS training initiatives, two of which were training courses at the Armed Forces Research Institute of the Medical Sciences (AFRIMS) in Thailand. The first included a two-hour mini-course, presented to 24 project managers, on the use of GIS and remote sensing, or the acquisition of information using either recording or real-time sensing devices, in vector-borne disease studies. USUHS conducted a second one-day course on using global positioning systems in the field and in GIS to 21 technicians.

In addition, USUHS continues to offer an introduction to GIS course as part of its master’s degree program in public health. In fiscal 2009, four students enrolled in this course. The University has also provided basic GIS training on an individual basis. University instructors provided basic GIS training to a visiting Thai student, whose dissertation involved examining the distribution of mosquito larvae in Southeast Asia, as well as an analyst in Korea.

Finally, USUHS administered a more advanced two-day course on ecological niche modeling to 17 GIS analysts at AFRIMS. This course focused on creating predictive maps of disease and vector distribution. The use of GIS in public health and epidemiology is an important tool, and the GIS training initiatives of USUHS provide public health students and lab personnel with important skills directly applicable to their field.

## U.S. Naval Medical Research Center Detachment (NMRCD)

Like the other overseas laboratories, NMRCD has a long and successful history of conducting innovative training in laboratory methods, epidemiology and research ethics. Each scientific department conducts laboratory training in their respective fields, but the Public Health Training Program within the Emerging Infections Department undertakes the majority of NMRCD training. This group has successfully leveraged U.S. Agency for International Development, CDC and the U.S. National Institutes of Health (NIH)/Fogarty International Center grants, in addition to AFHSC-GEIS funding, to further their training mission in the epidemiology of emerging infectious diseases.

The detachment has used multiple formats to accomplish its training mission. Target audiences included personnel from local Ministries of Health, Agriculture and Defense, academic staff and scientists from Peru and other countries in the Americas, as well as U.S. military and civilian scientists. The countries reached by NMRCD training included Chile, Paraguay, Peru and the United States.

The general approach to training at NMRCD promoted the integrated use of epidemiological, public health and clinical skills, coupled with innovative methods adapted specifically for the region of the Americas. Specifically, this included the continuation of a well-regarded outbreak investigation course in Peru and Paraguay, the introduction of a new course on field epidemiology methods in Peru, and the continued support for the fifth iteration of the NIH’s Clinical Research videoconference over three months.

## Challenges encountered by partners in conducting training initiatives

Although the majority of AFHSC-GEIS partners did not report challenges in conducting training initiatives, some did encounter difficulties. The most frequent problems noted by overseas partners related to host-nation issues, including political instability and insufficient infrastructure. For example, after the disputed Kenyan presidential elections in December 2007, the ensuing political instability and breakdown of law and order resulted in a burglary at USAMRU-K, where many AFHSC-GEIS program computers were stolen or vandalized. Likewise, political instability in Honduras impeded Public Health Command Region-South personnel from working effectively in the country for several months.

Insufficient host-nation infrastructure reported by AFHSC-GEIS overseas partners included unreliable communications networks and frequent power outages. Other reported challenges of undertaking AFHSC-GEIS projects outside the United States involved acquiring supplies, materials and equipment; finding and training competent personnel; establishing and maintaining collaborative agreements with host countries; and overcoming language barriers. Inadequate English proficiency was the most significant barrier to student performance in the University of Iowa’s certificate program in emerging infectious disease epidemiology. Funding delays, as well as the challenge of maintaining continuity of projects with uncertain funding, were also noted by several AFHSC-GEIS partners.

## Discussion

Experts note a growing realization that to improve global public health substantially, a training agenda must be developed that will sustainably educate the public health work force. Numerous training efforts now exist, but more comprehensive and coordinated approaches are needed. Some of these current efforts are extremely robust, such as the Field Epidemiology and Laboratory Training Program (FELTP), long considered the gold standard in public health training. However, this program reaches limited numbers of public health officials due to its comprehensive nature [12]. To fill this gap, shorter-term field, laboratory and academic training options are needed [13,14]. These directed training opportunities allow for the rapid acquisition of specialized skills, especially in laboratory techniques, outbreak investigation, and response and control of emerging infectious disease. Importantly, they serve a key role in supporting the implementation of the IHR (2005) and in meeting the local and regional public health needs of developing countries.

Epidemiologic and laboratory training are key components of public health capacity building, and thus, represent an important strategic goal of the AFHSC-GEIS network. From September 2008 through October 2009, the AFHSC-GEIS network, through 18 partner organizations, conducted 123 training initiatives in 40 countries that reached 3,130 U.S. and host-country personnel. Expanded training activities in the areas of pandemic preparedness, outbreak investigation and response, EID surveillance, and pathogen diagnostic techniques have contributed to building robust capabilities at the local level, in direct support of the implementation of IHR (2005).

In addition to documenting the educational initiatives of AFHSC-GEIS partners, this review provides recommendations for improving training activities that merit attention. The first and second recommendations are “global” in nature, requiring action by entities outside AFHSC-GEIS. The third and fourth recommendations are internal to AFHSC-GEIS, relating to quality improvement. Finally, the fifth recommendation requires the action of other entities, as well as AFHSC-GEIS itself.

**First, coordinate training initiatives among partners to avoid unnecessary duplication and maximize resources to address gaps.** This should be done with other state and non-state entities (CDC and WHO), as well as nongovernmental organizations, academia and other groups involved in global public health education. Training is an essential but time-consuming activity. Training conducted by AFHSC-GEIS partners in fiscal 2009 appear to be largely project-specific and do not seem to be closely coordinated within regions or between partners. As shown in Figure 1, respiratory infections (namely influenza) represented the majority of training initiatives (68 percent) in fiscal 2009. Although these educational efforts targeting influenza were important, especially in light of the recent pandemic, increased coordination and communication among partners may have brought focus to gaps in training, such as in sexually transmitted infections, one of the AFHSC-GEIS pillars (Figure 1).

**Second, plan and develop training initiatives in a strategic, systems manner.** A strategic and systematic approach to identifying training needs and developing educational initiatives would enhance coordination of efforts, assist in identifying appropriate subject matter experts and lessen the burden on any single organization. Such an approach could allow for increased efficiency and reduce duplication of efforts. Several efforts are under way to catalog or map education efforts, with the intention of fostering collaboration. The efforts include the AfriHealth project and WHO’s Knowledge Management for Public Health [15].

**Third, enhance the involvement of central headquarters in coordinating and supporting training initiatives.** Increased central coordination and scientific direction for AFHSC-GEIS projects can make existing training activities more efficient, productive and visible. Individual project progress may also benefit from more frequent scientific guidance. Given its ability to set policy and priorities for the global AFHSC-GEIS network and to interface with other interagency partners, AFHSC-GEIS is in a unique position to provide coordination and support of training initiatives.

**Fourth, establish a central repository of training materials used in AFHSC-GEIS-funded training initiatives.** A central repository of presentation slides, handouts, exercises and information on laboratory training kits would give partners a starting point from which to plan and coordinate their training activities.

**Finally, implement evaluation metrics, including measurements of learning objectives and competencies (i.e., pre- and post-tests, self-assessments and learner satisfaction).** Evaluation of the training initiatives is perhaps one of the most important steps. Simple pre- and post-tests could greatly enhance the efficiency and effectiveness of the training initiatives and identify the types of training that are the most successful and meaningful to the host country. Assessing the quality of training through a monitoring and evaluation framework across training programs, as well as assessing whether the level of sophistication is appropriate for the target audience, would be helpful.

The widespread realm of topics and geographic regions covered in the AFHSC-GEIS-funded training initiatives build on the center’s objective of worldwide emerging infectious disease surveillance and response. The training initiatives mentioned in this review all fall directly under the focus areas and targeted efforts of the IHR (2005) in developing the core competencies to identify, respond to and control public health threats and potential public health emergencies of international concern around the world. These AFHSC-GEIS-funded training initiatives serve as a conduit for member states and neighboring countries to communicate and collaborate on emerging infectious disease planning and development of their national strategies for addressing complex public health emergencies. Funding of training initiatives that enhance global emerging infectious disease planning and surveillance will greatly benefit the world’s population by preparing member states to address emerging infectious disease outbreaks in the future.

## Competing interests

To the best knowledge of the authors, no competing interests are reported.

## Supplementary Material

Additional file 1 **AFHSC-GEIS Funded Training Initiatives, September 2008-October 2009** * Training Modality Legend: W = Workshop, A = Academic Course, C = Conference, T = Tabletop exercise, D = Distance Learning, P = Telephone **†** Where exact figures are not known, an estimate of the number of trainees is provided. Click here for file
