# Supplementary figures and images for: Training initiatives within the AFHSC-Global Emerging Infections Surveillance and Response System: support for IHR (2005)
Source: BMC Public Health. 2011 Mar 4;11(Suppl 2):S5. doi: 10.1186/1471-2458-11-S2-S5 (PMC3092415; doi:10.1186/1471-2458-11-S2-S5)

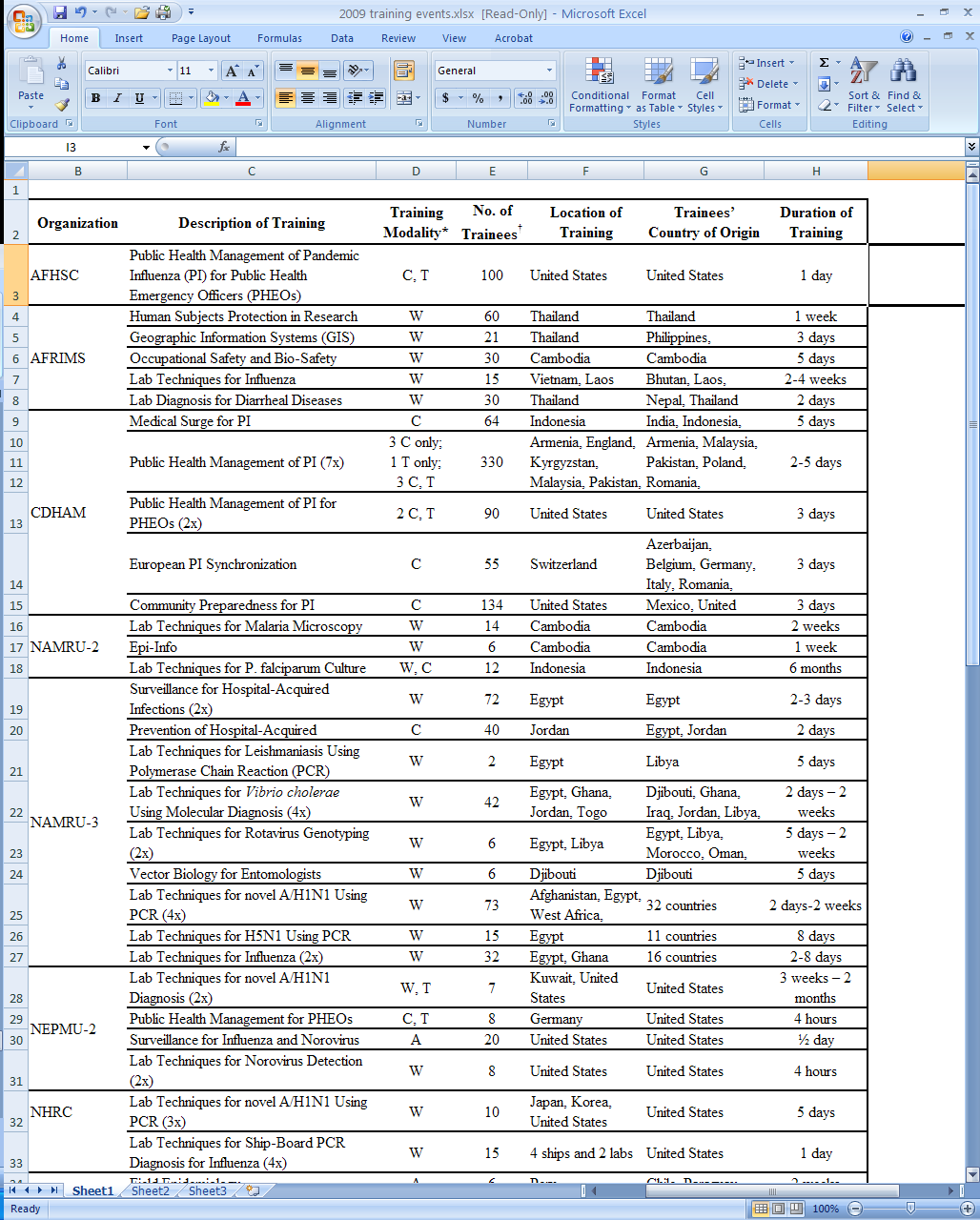


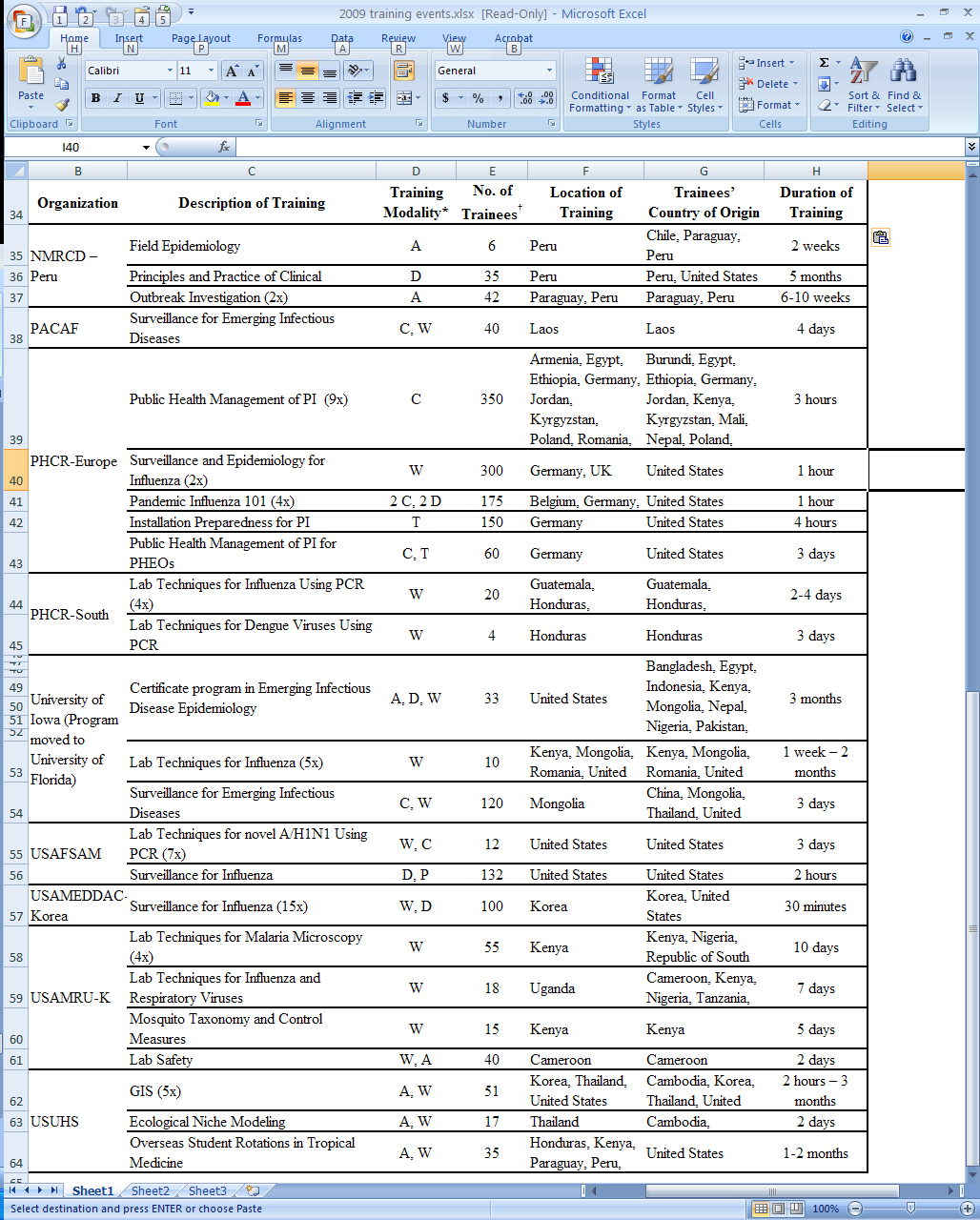

Supplement: Additional file 1 — AFHSC-GEIS Funded Training Initiatives, September 2008-October 2009 * Training Modality Legend: W = Workshop, A = Academic Course, C = Conference, T = Tabletop exercise, D = Distance Learning, P = Telephone † Where exact figures are not known, an estimate of the number of trainees is provided. [file 1471-2458-11-S2-S5-S1.docx]
